
JOURNAL INFORMATION
==============================
NLM Title Abbreviation: ChemistryOpen
Journal ID: ChemistryOpen
NLM Journal ID: 101594811
Journal ID: open

ChemistryOpen

EISSN: 2191-1363
Publisher: Wiley

ARTICLE INFORMATION
==============================
PMCID: PMC3594585
PMID: 24551522
DOI: 10.1002/open.201390000
Article version: 1
Subjects: News

Spotlights on our sister journals: ChemistryOpen 1/2013

Print publication date: 2013 Feb
Electronic publication date: 2013 Feb 27
Volume: 2
Issue: 1
First page: 10
Last page: 12
Copyright: Copyright © 2013 WILEY-VCH Verlag GmbH & Co. KGaA, Weinheim
Copyright year: 2013
License: Re-use of this article is permitted in accordance with the Creative Commons Deed, Attribution 2.5, which does not permit commercial exploitation.
License URL: https://creativecommons.org/licenses/by/2.5/

==============================
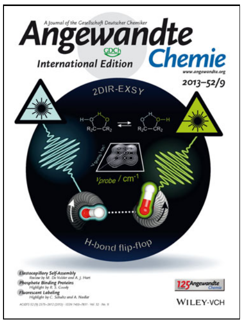

### Conjugated Polymers

*W. Han, M. He, M. Byun, B. Li, Z. Lin**

**Large-Scale Hierarchically Structured Conjugated Polymer Assemblies with Enhanced Electrical Conductivity**

**Stripes on a plane**: A set of highly ordered microscopic stripes (purple; see scheme) were produced over a large area using controlled evaporative self-assembly in a cylinder-on-Si geometry of conjugated homopolymers or all-conjugated diblock copolymer (P3BHT). The crystallinity of the as-prepared assemblies of P3BHT was greatly improved following chloroform vapor annealing, resulting in a fourfold increase in electrical conductivity.

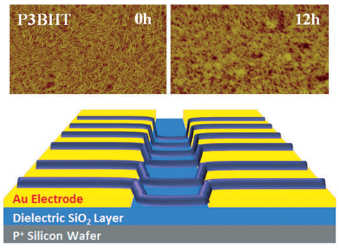

*Angew. Chem. Int. Ed.*
DOI: **10.1002/anie.201209632**

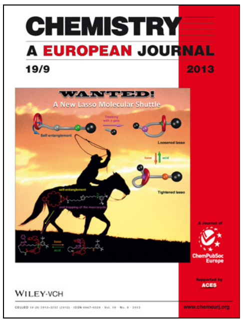

### Polyoxometalates

*R. S. Winter, J. Yan, C. Busche, J. S. Mathieson, A. Prescimone, E. K. Brechin, D.-L. Long, L. Cronin**

**Nanoscale Control of Polyoxometalate Assembly: A $\{Mn_8W_4\}$ Cluster within a $\{W_{36}Si_4Mn_{10}\}$ Cluster Showing a New Type of Isomerism**

**Nanoassembly**: Assembly isomers of a $\{Mn_8W_4\}$ Keggin cluster within a $[W_{36}Mn_{10}Si_4O_{136}(OH)_4(H_2O)_8]^{24-}$ cluster is reported. The clusters are formed through the aggregation of new $\{\gamma\text{-}SiMnW_9\}$ fragments in a tetrahedral fashion. Investigation of the crystal growth of these clusters revealed two intermediate clusters, which unveiled a mechanism of formation (see scheme). Physical analysis of the magnetic and solution behaviour of the isomers is also reported.

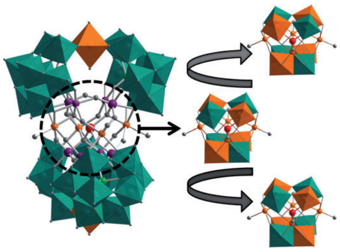

*Chem. Eur. J.*
DOI: **10.1002/chem.201204345**

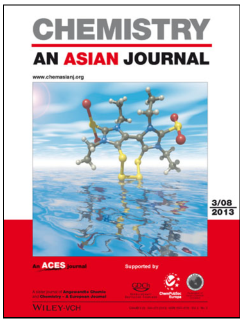

### Two-Photon Absorption

*T. He, Z. B. Lim, L. Ma, H. Li, D. Rajwar, Y. Ying, Z. Di, A. C. Grimsdale,* H. Sun**

**Large Two-Photon Absorption of Terpyridine-Based Quadrupolar Derivatives: Towards their Applications in Optical Limiting and Biological Imaging**

**Quadrupolar leap**: Two-photon absorption (TPA) properties of five novel terpyridine-based quadrupolar derivatives have been investigated to clarify the influences of molecular structures and molecular environment on their nonlinear optical behaviors in organic solvents and aqueous media. We found that alkylcarbazole as the donor and bis(styryl)benzene as a conjugation bridge can enhance TPA cross-sections of chromophores in both organic solvent and aqueous media.

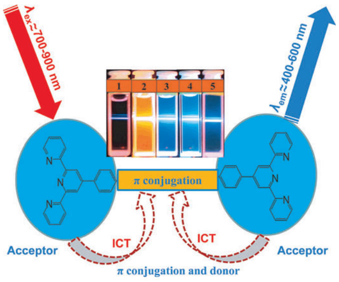

*Chem. Asian J.*
DOI: **10.1002/asia.201201009**

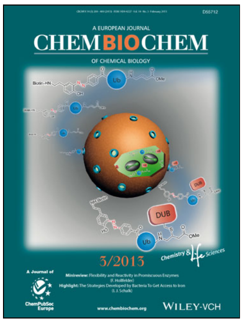

### Biosynthesis

*M. Kunert, P. Rahfeld, K. H. Shaker, B. Schneider, A. David, K. Dettner, J. M. Pasteels, W. Boland**

**Beetles Do It Differently: Two Stereodivergent Cyclisation Modes in Iridoid-Producing Leaf-Beetle Larvae**

**Defensive circles**: Leaf-beetle larvae synthesise the iridoid monoterpene chrysomelidial as a defensive compound. Feeding experiments with the deuterated precursor $[^2H_5]$8-hydroxygeraniol indicated two stereodivergent cyclisation modes towards chrysomelidial. To study the influence of the cyclisation mode on the stereochemistry, the configurations of chrysomelidials from seven species of leaf-beetle larvae were determined.

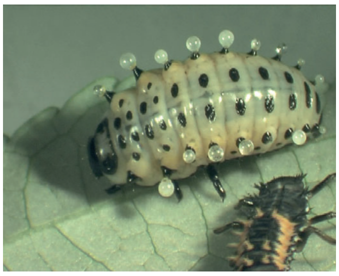

*ChemBioChem*
DOI: **10.1002/cbic.201200689**

WILEY ONLINE LIBRARY
© 2013 Wiley-VCH Verlag GmbH & Co. KGaA, Weinheim

### Photoelectrochemistry

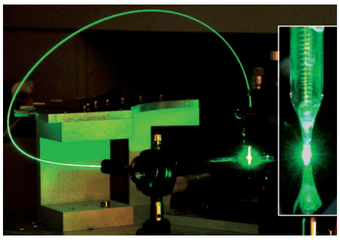

*J. P. Kollender, A. I. Mardare, A. W. Hassel**

**Photoelectrochemical Scanning Droplet Cell Microscopy (PE-SDCM)**

**Spot the drop**: Principles of localised photoelectrochemistry are summarised and an experimental approach, the photoelectrochemical scanning droplet cell microscopy (PE-SDCM, see picture), is described which allows the performance of the most important photoelectrochemical experiments within a diameter of 100 μm. Its general applicability is demonstrated by investigating n- and p-doped Si.

*ChemPhysChem*
DOI: **10.1002/cphc.201200656**

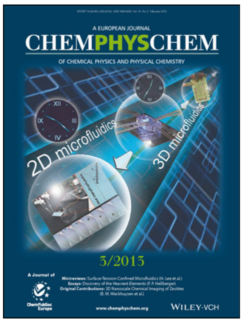

### Antiviral Agents

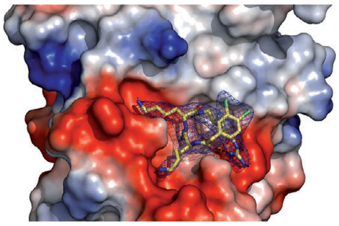

*M. Z. Hammamy, C. Haase, M. Hammami, R. Hilgenfeld, T. Steinmetzer**

**Development and Characterization of New Peptidomimetic Inhibitors of the West Nile Virus NS2B–NS3 Protease**

**Crystal clear**: A series of new substrate analogue inhibitors of the WNV NS2B–NS3 protease with an improved selectivity profile was developed, which contain decarboxylated arginine mimetics at the P1 position. For one of the most potent inhibitors, a crystal structure in complex with the WNV protease was determined.

*ChemMedChem*
DOI: **10.1002/cmdc.201200497**

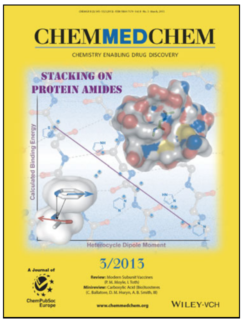

### Solar Cells

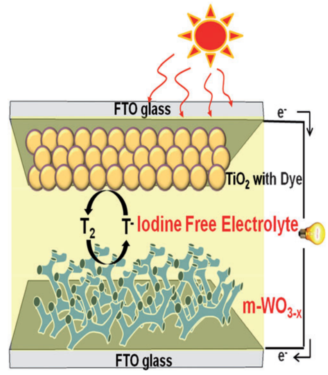

*I. Jeong, C. Jo, A. Anthonysamy, J.-M. Kim, E. Kang, J. Hwang, E. Ramasamy, S.-W. Rhee, J. K. Kim, K.-S. Ha, K.-W. Jun, J. Lee**

**Ordered Mesoporous Tungsten Suboxide Counter Electrode for Highly Efficient Iodine-Free Electrolyte-Based Dye-Sensitized Solar Cells**

**$WO_x$ before Pt**: Ordered mesoporous tungsten suboxide (m-$WO_{3-x}$) is synthesized by using a hard template and a partial reduction process. The m-$WO_{3-x}$ is used as an alternative catalyst for a counter electrode (CE) instead of Pt for iodine-free electrolyte ($T_2/T^-$)-based dye-sensitized solar cells. The catalytic activity of m-$WO_{3-x}$ CE is superior to that of conventional Pt CEs.

*ChemSusChem*
DOI: **10.1002/cssc.201200647**

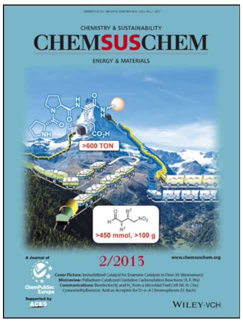

### Zeolites

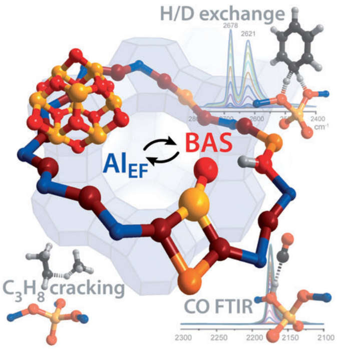

*S. M. T. Almutairi, B. Mezari, G. A. Filonenko, P. C. M. M. Magusin, M. S. Rigutto, E. A. Pidko, E. J. M. Hensen**

**Influence of Extraframework Aluminum on the Brønsted Acidity and Catalytic Reactivity of Faujasite Zeolite**

**Paraffin cracking** by Brønsted acid sites (BAS) in faujasite zeolites is strongly influenced by the presence of nearby extraframework aluminum ($Al_{EF}$) species (see picture). Although enhanced acidity of faujasites with decreasing framework Al density is directly evident from $CO_{ads}$ FTIR spectroscopy, H/D exchange reactions, and paraffin cracking activities, such correlations are less evident for $Al_{EF}$ modified zeolites, which is also discussed in terms of structure.

*ChemCatChem*
DOI: **10.1002/cctc.201200612**

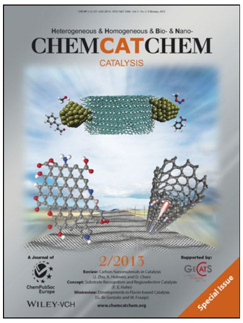

© 2013 Wiley-VCH Verlag GmbH & Co. KGaA, Weinheim

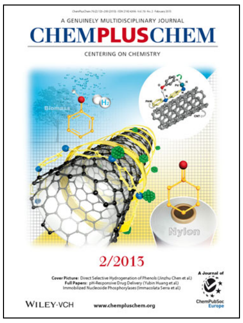

### Radical-Functionalised Gel

*M. Mannini, S. Cicchi, D. Berti, A. Caneschi, A. Brandi, L. Lascialfari,* L. Sorace**

**Radical-Functionalised Gel: A Building-Block Strategy for Magnetochiral Assembly**

**A radically new gel**: AFM and ESR spectroscopic studies are used to investigate a radical-functionalised gel and the gelation process (see graph). The analyses also provide evidence that the fibrillar structure is maintained below the gelling concentration. This opens relevant perspectives in terms of the realization of complex supramolecular chiral structures incorporating paramagnetic functions.

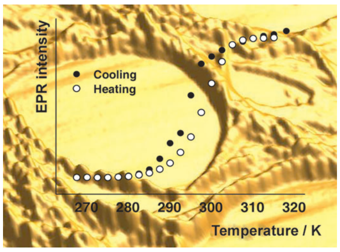

*ChemPlusChem*
DOI: **10.1002/cplu.201200250**

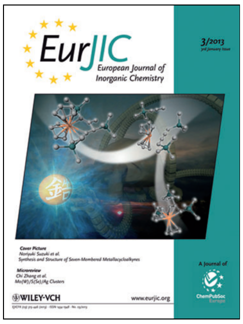

### Spin Crossover

*G. A. Craig, J. S. Costa, O. Roubeau, S. J. Teat, G. Aromí**

**An Fe$^{II}$ Spin-Crossover Complex Becomes Increasingly Cooperative with Ageing**

Improving with ageing! A bpp-type [bpp = bis(pyrazol-3-yl)pyridine)] ligand, $H_4L$, designed to enhance intermolecular interactions, leads to the spin-crossover (SCO) compound $[Fe(H_4L)_2](ClO_4)_2{\cdot}2THF{\cdot}H_2O$, which exhibits enhanced spin transition cooperativity with ageing. The intermolecular

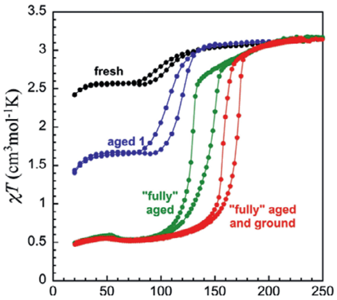

*Eur. J. Inorg. Chem.*
DOI: **10.1002/ejic.201201041**

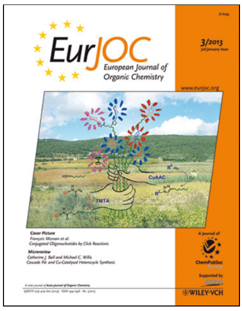

### Porphyrinoid Photosensitizers

*M. M. Bastos, A. T. P. C. Gomes, M. G. P. M. S. Neves, A. M. S. Silva, O. A. Santos-Filho, N. Boechat,* J. A. S. Cavaleiro**

**Synthesis of β-Substituted Porphyrin Derivatives Containing Heterocyclic Moieties as Potential Photosensitizers Against Cutaneous Leishmaniasis**

This publication reports the synthesis of porphyrin derivatives bearing aminoheterocyclic moieties at a β position. Molecular modeling and docking calculations show that all compounds synthesized have high affinity values to leishmanial arginase. These derivatives are potential leishmaniasis agents.

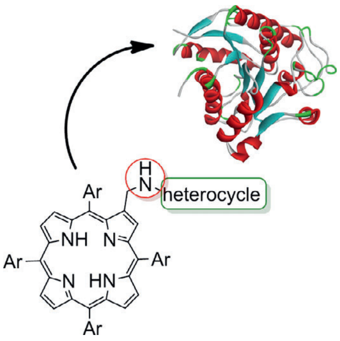

*Eur. J. Org. Chem.*
DOI: **10.1002/ejoc.201201501**

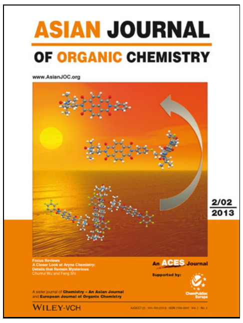

### Fluorescein Derivatives

*X. Xiong, F. Song,* S. Sun, J. Fan, X. Peng**

**Red-Emissive Fluorescein Derivatives and Detection of Bovine Serum Albumin**

**Red light**: Two fluorogenic compounds were synthesized by derivatization of 2′,7′-dichlorofluorescein at the C4′ and C5′ positions. These compounds have excellent photostability and emit at red wavelengths. Both compounds are highly selective for detecting bovine serum albumin because of their particular dyad structures.

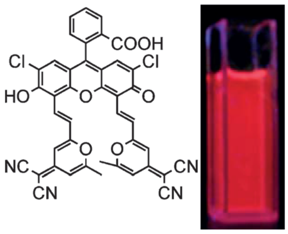

*Macromol. Rapid Commun.*
DOI: **10.1002/ajoc.201200109**

© 2013 Wiley-VCH Verlag GmbH & Co. KGaA, Weinheim
